# Poisoning by Arsenic

**Published:** 1848-01

**Authors:** 


POISONING BY ARSENIC.

A case was tried before the Jefferson Criminal Court, at its last term, of an attempt to poison by arsenic. We have been promised by the attending physician a full history of the case.

